# Respiratory Strength Training Versus Respiratory Relaxation Training in the Rehabilitation of Physical Impairment, Function, and Return to Participation After Stroke: Protocol for a Randomized Controlled Trial

**DOI:** 10.2196/59749

**Published:** 2024-11-27

**Authors:** Dorian K Rose, Gina Brunetti, Kathryn Cavka, J Brooke Hoisington, Hannah Snyder, Wei Xue, Barbara K Smith

**Affiliations:** 1 Department of Physical Therapy University of Florida Gainesville, FL United States; 2 Brooks Rehabilitation Jacksonville, FL United States; 3 Brain Rehabilitation Research Center Malcom Randall VAMC Gainesville, FL United States; 4 Department of Biostatistics University of Florida Gainesville, FL United States

**Keywords:** stroke, rehabilitation, exercise, clinical trial, respiration, wellness, community based

## Abstract

**Background:**

Persistent disability in chronic stroke survivors is often attributed to arm or leg weakness; however, respiratory muscle weakness also impedes poststroke rehabilitation, reduces quality of life, and increases the risk of health complications. Respiratory complications are common after stroke and place patients at risk for both prolonged functional disability and mortality. In addition, stroke survivors face ongoing cardiovascular disease that places them at risk for recurrent stroke.

**Objective:**

The study aims to compare the effects of 2 respiratory training programs, paired with individualized flexibility, strengthening, and cardiovascular exercise programs, on physiologic, activity, and societal participation outcomes in chronic stroke survivors.

**Methods:**

This study will be a randomized controlled trial. Participants are 80 community-dwelling adults with chronic stroke. In conjunction with a 24-session (3 times/week for 8 weeks), American Heart Association–informed, whole-body exercise program, participants will be randomized to receive either respiratory strength training or respiratory relaxation training. Study intervention will be directed by a physical therapist and take place in a community fitness center. Outcome assessments will occur in a clinical research center. The primary outcome measure is maximal respiratory pressure. Secondary outcome measures include airway clearance, walking endurance, spatial-temporal gait characteristics, community walking, functional strength and fatigue, depression, and societal participation measures. Longer-term societal participation is a complex domain that may be influenced by other factors beyond physical function. Participants’ health status will be monitored for 1 year following the intervention for falls, respiratory illness, and hospitalizations. Additional subanalyses will evaluate the effect of smoke exposure on short- and long-term outcomes. Outcome assessors are blinded to group assignments. Respiratory relaxation training is an active comparator, but no pure control group is included.

**Results:**

This study was funded in March 2020 with enrollment commencing in November 2020. Completion of enrollment is projected for May 2025 with a study projected end date of April 2026. Published results are anticipated in Fall 2026.

**Conclusions:**

Results from this study will improve our understanding of the additive benefits of respiratory exercises on short- and long-term physiologic, functional, and societal gains for these individuals. These data will be instructive to meet a current unmet rehabilitative need to promote patient-centered care and contribute to decreasing morbidity and mortality in chronic stroke survivors.

**Trial Registration:**

ClinicalTrials.gov: NCT05819333; https://clinicaltrials.gov/study/NCT05819333

**International Registered Report Identifier (IRRID):**

DERR1-10.2196/59749

## Introduction

### Background

Stroke is the leading cause of serious long-term disability in the United States [1]. A decrease in stroke mortality (36.9% decrease from 1999 to 2009), has led to a subsequent increase in those living with the consequences of stroke [2]. As such, it impacts the financial status of patients, families, and health care systems [3]. A retrospective study of inpatient stroke care costs from 2000 to 2020 revealed mean hospital costs per stay were US $15,781 (SD US $330) [4]. The mean lifetime cost of ischemic stroke is approximately US $140,048 in the United States, placing stroke among the top 10 most costly conditions among Medicare beneficiaries [5]. Recurrent stroke, cardiac disease, and respiratory complications are the leading causes of mortality in stroke survivors. Given these financial and health costs, there is therefore a critical need to prevent further cardiovascular and cardiorespiratory decline and reduce the incidence of cardiac and respiratory disease and functional disability.

Although stroke sequelae affect numerous body systems, automatic control of resting ventilation is typically spared in the majority of stroke cases, affected only in more severe brainstem strokes. Motor deficits are often the most debilitating to mobility and return to participation in life roles. Motor processing and the voluntary control of muscles of respiration engaged when speaking, upon exertion, coughing, or with exercise may be weakened following damage directly to the cerebral cortex or the descending axonal projections. Consequently, individuals after stroke may exhibit prolonged exercise intolerance, dyspnea, and airway clearance dysfunction. It is upon this conceptual framework we propose that respiratory muscle training is an important component in the rehabilitation of individuals following stroke. The purposes of this study are to (1) evaluate the effect of a combined exercise program (EP) with respiratory strength training (RST) on measures of impairment, activity, and participation; (2) evaluate its ability to maintain prospective respiratory health; and (3) explore the effect smoking history may have on these outcomes.

### Study Objective and Aims

The primary objective of this trial is to test the hypothesis that a combined EP with RST improves physiologic, activity, and societal participation outcomes in chronic stroke survivors. In addition to limb and respiratory weakness, up to 78% of patients after stroke present with dysphagia [6], a significant risk factor for aspiration. Cough is a critical mechanism to guard against aspiration and is often impaired after stroke [7], resulting in greater incidences of aspiration and chest infection [8,9]. Furthermore, pneumonia is the leading cause of nonvascular death in acute [10] and chronic [11] phases after stroke. RST could improve airway clearance as a protective function. The adoption of interventions capable of preventing the occurrence of respiratory complications may substantially improve the long-term outcomes of these patients [12]. Several small-scale studies have demonstrated that inspiratory [13-16] and expiratory [14] muscles respond to strength training in patients after stroke, but the impacts of respiratory strengthening on function are less clear. In a systematic review of poststroke respiratory muscle training randomized controlled trials, Menezes et al [17] reported there were insufficient data to determine whether the benefits of respiratory muscle training are realized in gains in functional activity level or participation, concluding that future trials are needed to assess the effects of training on one’s activity and participation in life roles. RST for individuals after stroke has the potential to facilitate a return to these important societal roles.

Our secondary objective is to prospectively monitor health status for 1-year following the exercise intervention. There is a clear link between the presence of respiratory dysfunction in individuals after stroke and the incidence of respiratory complications such as aspiration or respiratory infections [18-20]. Menezes et al [17] revealed that respiratory muscle training reduced the relative risk of respiratory complications immediately and 6 months after the commencement of the intervention. Others have reported that expiratory muscle strength training exercises improve respiratory strength, cough, and swallowing function in stroke [21-23]. A 2016 meta-analysis [17] revealed that respiratory muscle training reduced the relative risk of respiratory complications immediately and 6 months after the commencement of the intervention. However, this conclusion was based on just 2 trials, and procedures for detecting the occurrence of respiratory complications were not sufficiently robust.

An exploratory objective will evaluate potential differential responses to respiratory muscle training based on smoke exposure. Cigarette smoking negatively impacts respiratory health and increases the risk of cardiovascular events. While direct cigarette smoking is a known risk factor for stroke and poststroke complications, individuals exposed to secondhand smoke also face high health risks. There is a strong, dose-dependent association between passive smoking and stroke [24].

## Methods

### Trial Design

**Figure 1:**
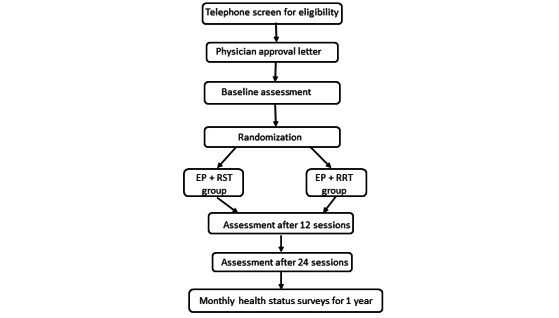
Flowchart of clinical trial. EP: exercise program; RRT: respiratory relaxation training; RST: respiratory strength training.

This is a single-blind randomized controlled trial. Participants will be assessed at Brooks Rehabilitation Clinical Research Center and receive intervention sessions at the Brooks Family YMCA. Health status will be monitored monthly for 1 year following the intervention. This study is registered at ClinicalTrials.gov (NCT05819333). The trial will be reported according to SPIRIT (Standard Protocol Items: Recommendations for Interventional Trials), TIDieR (Template for Intervention Description and Replication), and CONSORT (Consolidated Standards of Reporting Trials) guidelines (Figure 1).

### Ethical Considerations

This protocol and Informed Consent Form were reviewed and approved by the University of Florida’s institutional review board (IRB; #202000810). Participants will sign the IRB-approved informed consent form before enrollment. Participants will be assigned a unique numerical identifier to deidentify and assure the privacy and confidentiality of all collected data. Compensation of US $15/study visit will occur in the form of a debit card, with a total compensation of US $405.

To evaluate not simply how many steps participants take but to understand where those steps are taken (ie, within the confines of their home or actually outside their home in the community), participants will wear a GPS device for 7 days. These deidentified longitude and latitude coordinate data are not collected in real-time but rather will be downloaded to a password-protected computer at the completion of the 7-day wearing period. Safety of trial intervention and assessments will be monitored by reporting to the IRB any adverse events that occur from enrollment to trial completion at 12 months following the intervention.

### Participants

Participants will be recruited from an IRB-approved clinical research registry and study flier. Power calculations determined a study population of 80 participants and are presented below. We will enroll up to 95 participants to allow for a potential attrition rate of up to 15% (14/95). Specific inclusion and exclusion criteria for study enrollment are presented in Textbox 1. The cardiovascular and respiratory exclusion criteria were adopted from a previous exercise study with individuals poststroke [25,26].

**Inclusion and exclusion criteria for enrollment into the trial.**

**Inclusion criteria**

- Diagnosis of stroke >6 months after onset
- Signed letter of medical approval from primary care physician to participate
- Community dwelling
- Ability to attend the exercise program 3 times/week for 8 weeks
- Ability to follow directions or mimic exercises
- Ability to communicate adverse effects such as pain or fatigue or the need for assistance
- Able to ambulate 20 feet (6.1 m) with no more than contact guard assist, with or without an assistive device or orthotic device
- Able to access exercise equipment independently or with caregiver assist
- Older than 18 years of age

**Exclusion criteria**

- Neurologic conditions other than stroke (ie Parkinson disease or multiple sclerosis)
- Severe, functional limiting arthritis
- Orthopedic condition that limits mobility
- Severe weight-bearing pain
- Current participation in other physical rehabilitation services or exercise programs
- Serious cardiac conditions (hospitalization for myocardial infarction or heart surgery within the past year, history of congestive heart failure, documented serious and unstable cardiac arrhythmias, hypertrophic cardiomyopathy, severe aortic stenosis, angina or dyspnea at rest or during activities of daily living). Anyone meeting New York Heart Association criteria for Class 3 or Class 4 heart disease will be excluded.
- Severe hypertension with systolic >200 mm Hg and diastolic >110 mm Hg at rest, that cannot be medically controlled into the resting range of 180/100 mm Hg
- Use of supplemental oxygen
- Severe obstructive pulmonary disease (Classification of Global Initiative for Chronic Obstructive Lung Disease 3 or higher, indicating forced expiratory volume 1 <50% predicted)
- Treatment for pneumonia or lower respiratory infection within the past month
- Able to run one-quarter mile (402 m) without stopping

### Randomization, Allocation, and Blinding

Participants will be randomized to the EP plus RST group or EP plus respiratory relaxation training (RRT group) using the Microsoft Excel (Microsoft Office Professional Plus 2016) *RAND* function. Participants and the intervention physical therapist will be aware of the group assignment due to the nature of the intervention; however, study evaluators will remain blinded.

### Interventions

In total, 24 intervention sessions will be delivered 3 times a week over 8 weeks. Intervention therapists will undergo standardized training and a structured intervention log with the 3 aspects of the EP, and the respiratory training outlined will be followed each session to assure intervention fidelity.

### EP Components

Both groups will participate in the EP consisting of 3 components recommended by American Heart Association (AHA) poststroke exercise recommendations [27], directed by the intervention physical therapist, and individualized to the participants’ ability.

#### Strengthening Exercise

Resistance exercises will address primary muscle groups of the upper and lower limbs and trunk. Graded resistance will be provided through weight machines or resistance bands.

In terms of progression, the ability to perform an exercise with appropriate kinematics will determine initial resistance. Once the participant can perform 2 sets of 10 repetitions, resistance will be increased.

#### Cardiovascular Exercise

Exercise modalities may include treadmill, recumbent cross trainer, recumbent bicycle, stairs, elliptical stepper, upper-extremity ergometer, arc trainer or provided through overground gait, sit-to-stand repetitions, shallow knee bends, step-ups, and marching in place.

In terms of progression, participants will wear a heart rate monitor and exercise between 40% and 70% of heart rate reserve. The exertion will be subjectively reported using the rate of perceived exertion scale [28]. Progression will occur by increasing exercise duration, resistance, or speed.

#### Flexibility Exercise

Static stretching of the trunk and upper and lower extremities, 2 repetitions of each stretch held for 20 seconds. Muscles groups will be individualized according to participants’ needs.

### Respiratory Training

Participants will be randomized to the RST or RRT group.

#### RST Group

RST will consist of a bout of inspiratory strength training at the start of each EP session, plus a bout of expiratory strength training at the conclusion of each EP session. RST devices (Orygen Dual Valve, Forumed SL) provide up to 70 cm H_2_O of inspiratory and 80 cm H_2_O of expiratory pressure threshold loads. RST bouts include 5 sets of 5 maximal volume and speed breaths of both inspiratory and expiratory muscle training 2 times during each session. The initial intensity will be 25% of the maximal inspiratory and expiratory pressures the subject can generate. In terms of progression, the inspiratory setting of the training device will be increased up to 10% of the training load from the first set each session, to achieve a rate of perceived exertion of 6-8 (Modified 10-point Borg scale) [29] with complete valve opening. Expiratory settings will be progressed similarly.

#### RRT Group

RRT will consist of bouts of relaxation breathing at the start and conclusion of each EP session. Participants will be guided to breathe slowly through a respiratory training device (Threshold PEP, Respironics), with minimal resistance. RRT bouts include 5 sets of 5 breaths, 2 times during the session. While the effects of relaxation breathing exercises may include a modest lowering of systolic blood pressure in some hypertensive patients [30], this group serves as an active control. In terms of progression, there will be no progression for this group.

### Outcome Measures

Participants will be assessed (1) at baseline, (2) after 12 sessions, and (3) after 24 sessions by a physical therapist blinded to group assignment. Blinded assessors will undergo standardized training to assure assessment fidelity. Following intervention completion participants will complete monthly health status questionnaires.

#### Primary Objective Outcomes

**Figure 2:**
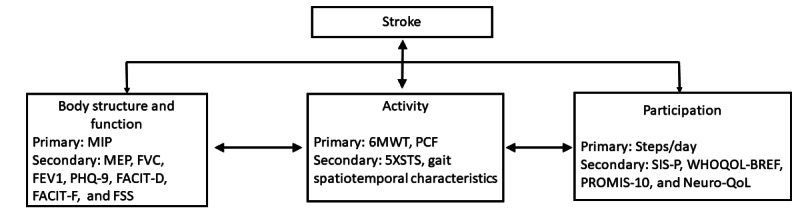
Outcome measures across World Health Organization’s International Classification of Functioning, Disability and Health model. 5XSTS: Five Times Sit to Stand; 6MWT: Six Minute Walk Test; FACIT-D: Functional Assessment of Chronic Illness Therapy – Dyspnea; FACIT-F: Functional Assessment of Chronic Illness Therapy – Fatigue; FEV1: Forced Expiratory Volume in 1 second; FSS: Fatigue Severity Scale; FVC: Forced Vital Capacity; MEP: Maximum Expiratory Pressure; MIP: Maximum Inspiratory Pressure; Neuro-QOL: Neuro Quality of Life; PCF: Peak Cough Flow; PHQ-9: Patient Health Questionnaire -9; PROMIS-10: Patient-Reported Outcomes Measurement Information 10; SIS-P: Stroke Impact Scale – Participation; WHO-QOL BREF: World Health Organization Quality of Life Brief Version.

The World Health Organization’s International Classification of Functioning, Disability, and Health model [31] recognizes that functioning and disability as a result of a health condition (stroke, in this trial) are multidimensional concepts, relating not simply to impairment at the level of one’s body (ie, muscle strength) but to limitations in activity one might experience, as well as one’s restrictions in societal participation. To assess more broadly how addressing respiratory muscle weakness can improve quality of life after stroke, we will examine outcomes across these dimensions (Figure 2).

Our primary outcome is maximum inspiratory pressure (MIP). MIP is a measure of the strength of inspiratory muscles, primarily the diaphragm [32]. Participants will perform a seated MIP maneuver using a respiratory pressure manometer, from residual volume, in accordance with American Thoracic Society testing guidelines for respiratory muscle testing [33]. Additional respiratory measures include maximal expiratory pressure (measure of the strength of expiratory muscles, primarily the abdominals and intercostals) [33], forced vital capacity (the total volume of air blown out of the lungs during forced exhalation after maximal inspiration) [33], forced expiratory volume in 1 second (a measure of the amount of air that can be forced out of the lungs in one second following a full inhalation) [33], and peak cough flow (maximum expiratory flow during the compressive phase of a cough) [34]. Open circuit spirometry will provide breath-by-breath cardiopulmonary data while participants complete the 6-minute walk test [35]. Additional measures of physical function include the 5-times sit-to-stand test [36] and spatiotemporal gait characteristics captured while walking across an instrumented walkway. Steps per day and GPS data will be obtained at baseline and intervention conclusion for 1 week. A portable accelerometer will record participants’ steps, and a GPS device will record location. A walk score [37,38] and an area deprivation index [39] score will objectively characterize the “walkability” and the socioeconomic status of the participant’s neighborhood.

Finally, self-report measures of participation and quality of life will also address our primary objective. These include the Participation subsection of the Stroke Impact Scale [40]; the abbreviated World Health Organization Quality of Life instrument [41], which measures the domains of physical health, psychological health, social relationships, and environment; the Functional Assessment of Chronic Illness Therapy Dyspnea [42] and Fatigue [43] Scales; the Fatigue Severity Scale [44]; the Patient Health Questionnaire-9 depression assessment [45]; the Patient Reported Outcomes Measurement Information-10, an assessment of health status after stroke [46]; and the satisfaction and ability to participate in life roles subscales of the Neuro Quality of Life [47] (Table 1).

**Table 1:** Outcome measures.

Assessment	Outcome measured	Time to complete	Scoring	Psychometrics
MIP^a^	Maximum pressure created during inspiration measured with a manometer	8 minutes	Pressure measured in cm H_2_O	Reliability [48] Validity [48]
MEP^b^	Maximum pressure created during expiration measured with a manometer	8 minutes	Pressure measured in cm H_2_O	Reliability [48] Validity [48]
PCF^c^	Strength of cough	5 minutes	Liters of air breathed out per minute; L/min	Reliability [49] Validity [49]
FVC^d^	Maximum amount of air exhaled after fully inhaling and making a maximal effort.	5 minutes	Volume measured in liters	Reliability [50,51] Validity [50,51]
FEV_1_^e^	The volume of air exhaled in the first second during forced exhalation after maximal inspiration	1 minute	Volume measured in liters	Reliability [50,51] Validity [50,51]
6MWT^f^	Participants walk for six minutes covering as much ground as possible. Participants were permitted to stop and rest at any time during the test.	6 minutes	Distance measured in meters	Reliability [52] Validity [53]
5XSTS^g^	Participants stand up and sit down from a 16” solid seat as quickly as possible 5 times, with their arms folded across their chest.	1 minute	Time is measured in seconds	Reliability [54] Validity [54]
Steps/day	Number of steps taken over a seven-day period measured with an activity monitor	7 days	Number of steps completed each day	Reliability [55] Validity [56]
PHQ-9^h^	Self-report questionnaire to assess depressive symptoms	6 minutes	Nine items scored on a 0-3 Likert scale	Reliability [57] Validity [57]
SIS-P^i^	Self-report questionnaire to assess ability to participate in meaningful life activities	3 minutes	Eight items scored on a 1-5 Likert scale	Reliability [58] Validity [58]
WHO-QOL BREF^j^	Self-report questionnaire to assess four QOL domains: physical health, psychological health, social relationships, and environment.	12 minutes	26 items rated on a 5-point Likert scale. Each domain score is transformed into a scaled score, with a higher score indicating a higher QOL.	Reliability [59] Validity [59]
FSS^k^	Self-report questionnaire to assess the impact of fatigue on life and functional activities	8 minutes	Nine items scored on a 1-7 Likert Scale; one item scored on a 0-10 visual analog scale.	Reliability [60] Validity [60]
FACIT-D^l^	Self-report questionnaire to assess dyspnea severity during activities of daily living	5 minutes	Ten items scored on a 0-3 Likert scale	Reliability [42] Validity [42]
FACIT-F^m^	Self-report questionnaire to assess fatigue experience and impact on daily life	5 minutes	Thirteen items scored on a 0-4 Likert scale	Reliability [61] Validity [61]
Neuro-QOL^n^	Two self-report questionnaire subscales to assess 1) the Ability to Participate in Social Roles and Activities and 2) Satisfaction with Social Roles and Activities	10 minutes	A total of 16 items scored on a 1-5 Likert scale	Reliability [47] Validity [47]
PROMIS-10^o^	Self-report questionnaire to assess multiple domains of health	5 minutes	Ten items scored on a 1-5 Likert scale	Reliability [62] Validity [62]

^a^MIP: maximum inspiratory pressure.

^b^MEP: maximum expiratory pressure.

^c^PCF: peak cough flow.

^d^FVC: forced vital capacity.

^e^FEV1: forced expiratory volume in 1 second.

^f^6MWT: 6-Minute Walk Test.

^g^5XSTS: Five Times Sit-to-Stand.

^h^PHQ-9: Patient Health Questionnaire-9.

^i^SIS-P: Stroke Impact Scale–Participation.

^j^WHO-QOL BREF: World Health Organization Quality of Life Brief Version.

^k^FSS: Fatigue Severity Scale.

^l^FACIT-D: Functional Assessment of Chronic Illness Therapy–Dyspnea.

^m^FACIT-F: Functional Assessment of Chronic Illness Therapy–Fatigue.

^n^Neuro-QOL: Neuro Quality of Life.

^o^PROMIS-10: Patient-Reported Outcomes Measurement Information 10.

#### Secondary Objective Outcomes

To investigate if RST in the chronic poststroke period impacts the rate of respiratory complications, we will follow participants for 1 year after intervention. Respiratory complications will be defined as the onset of respiratory symptoms that require the evaluation and treatment by a medical provider or hospitalization for respiratory causes. If the participant reports a respiratory complication, medical records will be obtained and a blinded assessor will review the record to confirm diagnosis.

#### Exploratory Objective Outcomes

To address this objective, participants will provide a urine cotinine test and complete a subset of key questions from the Global Adult Tobacco Survey, a validated self-report of tobacco consumption and passive smoking [63]. From the cotinine test results, participants will be categorized as either (1) no significant smoke exposure or (2) significant smoke exposure. Cotinine is a metabolite of nicotine and indicates exposure to tobacco products within the past 48 hours. The Global Adult Tobacco Survey is a household survey created as a way to globally monitor adult tobacco use and indirectly measure the impact of tobacco control and prevention initiatives. A subset of questions will be asked about current and past tobacco use (if any) and exposure to secondhand smoke. We will compare the outcome measures described above from our primary and secondary objectives to investigate differences between those categorized as no significant smoke exposure and significant smoke exposure.

### Data Analysis

#### Power Calculations

Our primary outcome measure is MIP. The power calculation was based on work by Messaggi-Sartor et al [14], who examined the effect of RST on respiratory strength and respiratory complications following subacute stroke and measured MIP as the primary outcome. MIP mean gain was 18.9 (SD 15.1) cm H_2_O in the experimental RST group and the mean MIP change was 9.3 (SD 10.1) cm H_2_O in the control (sham RST) group. With a total of 80 participants (40 in each group) in this study, a 2-sided repeated measures ANOVA comparing 2 treatments across 3-time points, using a pooled SD of 12.8 would detect a difference at power >0.8 (f=0.16, F=3.067, =0.5, =.5). For our secondary objective, the RST effect on lung infections may be even more robust. Messaggi-Sartor et al [14] reported a 70% lower lung infection rate for subjects who completed RST during subacute stroke rehab, compared with subjects allocated to the control group. The number needed to treat to prevent one lung infection event was seven [14]. The effect of RST on respiratory complications for those with chronic stroke is not yet known. Our exploratory objective is to distinguish responses to RST, based on smoking exposure. The primary intent of this objective is to collect data to power a future, larger study. Specifically, baseline function is lower in current smokers without neurological disease, and their tolerated exercise training volume may be reduced. A sample size of 80 will provide an opportunity to evaluate whether smoking influences responses to the exercise interventions.

#### Statistical Analysis

To analyze the data for each objective and investigate the intervention effects for EP+RST versus EP+RRT, we will adopt a mixed effects model [64] to account for the correlation of repeated measurements within each participant. Depending on the outcome for each objective, we will include the baseline value as an independent covariate in addition to other independent variables including intervention effects at 4 weeks and 8 weeks, age, gender, and BMI by using a robust covariance matrix, that is, unstructured covariance matrix.

## Results

This study was funded in March 2020 with enrollment commencing November 2020. As of July 2024, a total of 69 participants were enrolled. Completion of enrollment is projected for May 2025 with a study projected end date of April 2026. Published results are anticipated in Fall 2026.

## Discussion

### Anticipated Findings

Results from this study will improve our understanding of the additive benefits of respiratory exercises on short and long-term physiologic, functional, and societal gains for these individuals. We hypothesize that the combined RST+EP will be more effective than the EP alone for stroke survivors with and without a smoking exposure history. Specifically, we hypothesize that combined RST+EP will be more effective in restoring poststroke maximal respiratory pressure, in restoring both physical (walking endurance) and respiratory (airway clearance) function, and in restoring poststroke life and social roles. We also hypothesize that RST+EP will be more effective than EP alone in reducing 1-year postintervention incidence of adverse respiratory events.

### Wellness Approach

While multiple studies indicate the potential for respiratory muscle training to improve respiratory strength, airway protection, and dyspnea during acute stroke rehabilitation [14,65,66], limited information is available pertaining to the benefits of respiratory training in community-dwelling adults with chronic stroke [13,21,67]. This study approaches the research aims from a wellness perspective, as opposed to a medical model of rehabilitation. The AHA-informed EP and respiratory training sessions occur in a community-based gym that emphasizes wellness and secondary prevention of stroke. Previous respiratory training research has emphasized respiratory strength, with fewer studies reporting effects on activity-based metrics such as cough force, gait speed, or walking endurance [16,68]. This trial uses an impairment-based primary study outcome measure (MIP) as a comparator to other published respiratory training studies in stroke. However, activity- and participation-based outcome measures were also included to account for functional limitations participants might experience after stroke and to further quantify restrictions in their societal roles. Further, this study moves beyond patient-reported participation, by directly measuring impacts of the respiratory training on gains in community activity and societal roles for up to 1 year following the completion of the intervention. The carryover from a gain in body function or activity to improved community and societal participation is a recognized need in stroke rehabilitation research [17].

### Effects of Smoke Exposure

This research will also contribute novel exploratory information regarding effects of the EP and respiratory training in people with chronic stroke with and without daily cigarette smoke exposure. Smoking is an independent risk factor for stroke that directly increases the relative risk of stroke in a dose-dependent fashion [69]. Smoking induces vascular inflammation and endothelial dysfunction to accelerate the progression of atherosclerosis [70,71]. Secondhand smoke exposure also independently increases both the risk of stroke and obstructive pulmonary disease [72,73] and no level of passive smoke exposure is considered safe [24]. Chronic smoke exposure increases the work of breathing through dynamic hyperinflation and heightened airway resistance, despite an increased inspiratory neural drive, leading to dyspnea and airway clearance dysfunction [74,75]. Despite the negative impacts of smoking on cardiovascular and respiratory health, its impact on poststroke respiratory rehabilitation is poorly understood. Older adults with obstructive lung disease who completed 6 weeks of inspiratory strength training achieved significant improvements in MIP and dyspnea, and reported physical quality of life, yet in contrast to adults without lung disease, no gains in walking endurance occurred. These results suggest that individuals with lung disease recovering from stroke may require additional training time or combinatorial exercises to improve walking [76].

### Limitations

We recognize some limitations of the study design. The study was powered to distinguish the 8-week effects of RST from RRT on MIP. We acknowledge that societal participation is multidimensional, and longer-term changes may be influenced by many contributing factors beyond respiratory strength and walking endurance [77,78]. The use of patient-reported outcome measures and assessment of the Area Deprivation Index will help identify some factors that contribute to societal participation. Since group assignment will be based on the type of respiratory training, it is possible there may be unequal proportions of smoke-exposed and nonsmoking participants in each group. The monitoring of smoke exposure will enable us to explore whether the combined respiratory and whole-body exercise approaches offer distinct benefits to cigarette smoke-exposed individuals. While study assessors will be blinded to group assignment, patients and exercise supervisors will know their respiratory exercise assignment. A true control or “sham” respiratory training intervention was not included; rather, the RRT serves as an active comparator. The volume of respiratory exercises during exercise sessions will be the same for both groups, even though the physiological effects of RST and RRT exercises likely differ.

### Dissemination Plan

Since findings from this study may influence the health promotion, wellness, and secondary prevention societal roles of the physical therapy profession, dissemination efforts will be directed toward physical therapists and other rehabilitation providers. Study results will be presented at scientific conferences and published in peer-reviewed journals used by physical therapists and rehabilitation scientists.

### Conclusion

This trial is an investigator-initiated, single-blind, randomized controlled trial designed to evaluate the effects of an AHA-informed EP and respiratory training on measures of impairment, activity, and societal participation. Implementation of this trial will help identify modes of exercise that lead to the greatest short- and long-term functional and societal gains in smoke-exposed and nonexposed, community-dwelling adults with chronic stroke.

## Appendix

Multimedia Appendix 1 Peer-review report 1 from the Florida Department of Health James and Esther King Research Program 18-19 (USA).

Multimedia Appendix 2 Peer-review report 2 from the Florida Department of Health James and Esther King Research Program 18-19 (USA).

## Abbreviations

AHA: American Heart Association
CONSORT: Consolidated Standards of Reporting Trials
EP: exercise program
IRB: institutional review board
MIP: maximum inspiratory training
PROMIS-10: Patient Reported Outcomes Measurement Information 10
RRT: respiratory relaxation training
RST: respiratory strength training
SPIRIT: Standard Protocol Items: Recommendations for Interventional Trials
TIDieR: Template for Intervention Description and Replication

## Data Availability

All data generated or analyzed during this study, once available, will be included in the published results.
