# Supplementary material for: Respiratory Strength Training Versus Respiratory Relaxation Training in the Rehabilitation of Physical Impairment, Function, and Return to Participation After Stroke: Protocol for a Randomized Controlled Trial
Source: JMIR Res Protoc. 2024 Nov 27;13:e59749. doi: 10.2196/59749 (PMC11635318; doi:10.2196/59749)
Supplement: Multimedia Appendix 1 [file resprot_v13i1e59749_app1.pdf]

Review: Florida Department of Health James and Esther King Research Program 18-19

Proposal Number: 18-2033

Proposal Title: Augmenting a Post-Stroke Wellness Program with Respiratory Muscle Training: A Randomized Controlled Trial

Principal Investigator Name: Rose, Dorian

Significance:

Mean Score for Significance: 2

Strengths:

Reviewer 1:
The proposal addresses a highly prevalent health problem (stroke rehabilitation) and one that is in need of additional research. There has been very little research on the effects of respiratory muscle training in stroke patients. The significance is very much bolstered by the plan to conduct the study in a community-based setting and capitalizes on an existing relationship between a YMCA and a hospital. Finally, the outcomes to be assessed are important and patient-centered which will add to the health relevance and overall applied impact of the proposed research.

Reviewer 2:
While acute stroke treatments have decreased mortality after stroke, there is a concomitant increase in the number of stroke survivors that experience long-term disability. Many of these individuals have poor quality of life, increased morbidity, and high incidence of rehospitalization and recurrent strokes. Dysphagia and pneumonia are common conditions associated with long-term survival after stroke, especially in older populations. This study is significant because it assesses the effectiveness of a respiratory muscle training program to increase respiratory strength, lung health and functional mobility. The respiratory intervention is added to a stroke wellness program that focuses on general strengthening and cardiovascular health. Importantly, because smoking is an independent risk factor for stroke, the study will assess the effects of the intervention in smokers (and those exposed to second-hand smoke) and non-smokers. The results from this study are likely to have a major impact on our understanding of the effects of smoking on post-stroke outcomes, and the ability to alter the respiratory health and quality of life of both smokers and non-smokers with stroke.

Reviewer 3:
This study addresses important clinical question after stroke using combined wellness program and respiratory muscle training

Significance:

Weaknesses:

Reviewer 1:
None noted

Reviewer 2:
As a single-site study, the implications of the results will be limited. However, this is a novel pilot study that can be readily adapted to other facilities with cohorts of chronic stroke survivors.

Reviewer 3:
It is unclear if improvement in MIP will translate to improved outcome after chronic stroke which can be heterogeneous based on brain injury. In addition not all stroke patients will have impaired respiratory function. If so it is not clear what is the mechanism.

Investigator(s):

Mean Score for Investigator(s): 3

Strengths:

Reviewer 1:
Dr. Rose is highly qualified to lead this research based on her prior training and experience. She is supported by a collaborator (Dr. Smith) with experience in respiratory muscle training. There is no concern that these two can successfully conduct the trial and collect the data.

Reviewer 2:
The PI (Rose) is highly skilled at conducting rehabilitation trials in stroke patients and has a strong track record of very good to excellent publications. The co-I (Smith), while somewhat less experienced, has several recent publications demonstrating expertise in ventilatory muscle function. She currently is PI of an R21 on diaphragm pacing in Pompe Disease, and is a co-I on one other active grant (another ended in November). The biostatistician seems to have the training to assist in the conduct of this study.

Reviewer 3:
The PI and investigative team are well suited to do the study

Investigator(s):

Weaknesses:

Reviewer 1:
The minor concern about personnel is in the data management and analysis aspect. It does not appear that either Dr. Rose or Smith has worked or published with Dr. Zou in the past and there does not appear to be enough effort for his involvement, particularly in the later years of the project when the data analyses will take place. In addition, there is no description of how the data will be stored and managed over the course of the trial, questioning the early-career status and prior experience level of Dr. Zou.

Reviewer 2:
The biostatistician is relatively junior, and has relatively little experience with similar randomized trials. This weakness is considered relatively minor. The project coordinator is a graduate student, and may not have the appropriate experience and training for the coordinator role, especially with regard to IRB document preparation, training of study personnel, etc. In addition, it seems unusual to assign a graduate student to a full-time (0.50 FTE) position over five years. While more of a budgetary flag, it is confusing to have a 0.5FTE for a graduate assistant at >$30K.

Reviewer 3:
The PI has few publications as first or senior author in the area of the study

Innovation:

Mean Score for Innovation: 2.6667

Strengths:

Reviewer 1:
There are several highly innovative aspects of the proposed study, including the novel use of respiratory muscle training in a community-based stroke wellness setting, the significant and patient-centered outcomes to be assessed, and the exploratory long-term follow-up of the interventions. In addition, the hypothesis is novel and the data will provide preliminary insight into whether RMT enhances exercise training efforts and into whether outcomes differ by smoking status.

Reviewer 2:
While the respiratory muscle training is not entirely novel, the application to a chronic stroke population, especially distinguishing the effects in individuals with and without exposure to smoking, is highly innovative.

Reviewer 3:
The study proposed design and clinically oriented intervention.

Innovation:

Weaknesses:

Reviewer 1:
None noted

Reviewer 2:
No significant weaknesses.

Reviewer 3:
The study is uses standard respiratory muscle exercises therefore study techniques are not novel but the application in this group of patients is relatively new. In addition the study uses old version of devices for RMT. Newer version of digital devices with tracking ability are available.

Approach:

Mean Score for Approach: 4.3333

Strengths:

Reviewer 1:
The approach is focused and clear-cut and will be conducted in an appropriate manner to address the stated hypotheses. The plan to use blinded assessors is good, as well as the plan to use an active control that also does the relaxed breathing exercises. There is strong rationale for each of the 3 study aims and the methods for data collection are valid and reliable. The inclusion/exclusion criteria are appropriate and there are no concerns about study retention.

Reviewer 2:
The overall design of the study is excellent with sufficient intervention periods (3x/week for 8 weeks) to reasonably predict a change in respiratory function. Also, the longitudinal data is critical for translation of the results to potential clinical treatment. Inclusion/exclusion criteria are reasonable, and outcomes measures well-justified. Given that limited data is available on which to base a formal power analysis, the approach is reasonable.

Reviewer 3:
The study design as RCT is strong using combined technique for rehabilitation.

Approach:

Weaknesses:

Reviewer 1:
Concerns with the approach are based on the lack of details provided regarding the progression of the exercise training and the credentials of the staff who will be supervising the exercise sessions. This is not clear in the proposal. There is also no rationale provided for the 8-week timeframe of the intervention. Is it long enough to elicit improvements in respiratory muscles?
In addition, the sample size was not stated until the power analysis section and there is very little attention given as to how the 80 subjects will be recruited and who will do this. The accrual rate is quite slow and recruitment will continue into Year 5, leaving no time for the 1-yr follow-up of the final participants. It is not clear how many participants join the Stroke Wellness Program each year and where these patients are referred from. Can additional patients be referred to this program so that recruitment can be condensed?
The power analysis section is weak, but since this is a pilot study to collect data for a larger trial, this is a minor concern.

Reviewer 2:
The Fit-Bit Ultra has been tested for accuracy in step counts, but in relatively high-functioning ambulators with stroke. It is not entirely clear whether accuracy will be more limited in the study population. Also, individualized calibration of such step-trackers can improve accuracy, but this is not discussed. There is no discussion of a trial registry, which is essential for eventual publication of the data.

Reviewer 3:
The study lacks sufficient preliminary data to assure feasibility of the proposed study.
Data presented of improved MIP was from ICU patients who are mechanically ventilated. One case was presented with improvement in MIP by 6%. MIP and MEP measurements are very effort dependent and do not reflect ctural changes or outcome.
The power analysis and sample size estimation of the study is not very clear.

The inclusion criteria of chronic stroke survivors are heterogeneous and could prevent the study from being able to answer the question of the aims as response will be variable and lack mechanistic approach.

Environment:

Mean Score for Environment: 1.3333

Strengths:

Reviewer 1:
There are enough resources to conduct the study and the environment is exceptional

Reviewer 2:
Access to the Brooks Stroke Wellness program is an essential asset. Also, all of the equipment required to conduct the physiological assessments and intervention are in place.

Reviewer 3:
Strong environment

Environment:

Weaknesses:

Reviewer 1:
No concerns

Reviewer 2:
None.

Reviewer 3:
No weakness

Health Impact:

Mean Score for Health Impact: 3.3333

Strengths:

Reviewer 1:
The health impact of this line of research has the potential to be very strong. If it is shown that adding respiratory muscle training enhances health and well-being of stroke patients, this would be a simple adaptation to add to the rehabilitation therapies for stroke patients. In addition, the prevalence of stroke events and the debilitating nature of the event and subsequent lack of full recovery adds to the imperative nature of this research.

Reviewer 2:
The potential health impact for Floridians with stroke is highly significant. Stroke is prevalent in Florida, and burden of stroke complications related to smoking exposure is high. If such an intervention can be shown to be effective in smokers and non-smokers alike, a very large percentage of survivors will benefit from the research. If efficacy is demonstrated, rehospitalization will be reduced, and quality of life will be improved.

Reviewer 3:
The study employs practical approach that can be easily disseminated across communities and health care facilities.

Health Impact:

Weaknesses:

Reviewer 1:
None noted

Reviewer 2:
As this is an early-stage protocol, the intervention will be conducted at a single site. Generalization to a multi-site study in the future is not discussed. Also, it is not clear if such interventions would be efficacious (or even feasible) with home-bound individuals who may be in the most need of respiratory strengthening.

Reviewer 3:
It is not clear that the proposed outcome (e, g, MIP) and approach will lead to significant health impact

OVERALL IMPACT

Mean Score for Overall Impact: 3.3333

Strengths:

Reviewer 1:
This proposal will discover whether addressing respiratory muscle weakness in a community-based setting improves highly relevant outcomes of importance to stroke survivors. The study is designed with utmost rigor as a randomized, single-blind, trial with an active control group. The methods to collect the outcomes are valid and reliable and the investigative team is excellent and highly qualified. The strengths of this proposal highly outweigh the minor weaknesses noted.

Reviewer 2:
Potential for high impact on health of stroke survivors in Florida. Excellent PI and co-I with complementary experience to conduct the RCT. Outstanding facilities and availability of participants. Very good to excellent study design with longitudinal assessment along multiple ICF domains.

Reviewer 3:
Overall this is a well written proposal that addresses important and common health problem with high risk of disabilities and morbidities. The study design and investigative team are strong.

OVERALL IMPACT

Weaknesses:

Reviewer 1:
The minor concerns noted are the questionable expertise and lack of prior collaboration in the area of biostatistics and the slow recruitment plan. These are easily addressed and do not distract from overall high enthusiasm for the project.

Reviewer 2:
Questionable role of graduate student as project coordinator. Initial impact of results will be limited due to single-site design.

Reviewer 3:
The enthusiasm for the study was dampened by lack of conceptual frame for therapeutic impact after stroke, lack of preliminary data relevant to the proposed aims to assure feasibility, and ambiguous approach.

Protections for Human Subjects:

Description of any concerns and recommendations:

Reviewer 1:
None noted

Reviewer 2:
While PI will ultimately be responsible for oversight of human protection, there is some concern with utilization of an inexperienced graduate student with significant responsibilities for developing IRB documents.

Reviewer 3:
No concern

Vertebrate Animals:

Description of any concerns and recommendations:

Reviewer 1:
None noted

Reviewer 2:
Not applicable.

Reviewer 3:
NA

Budget and Period of Support:

Recommended modifications (if any):

Reviewer 1:
No modifications needed. The budget is very well justified

Reviewer 2:
It may be an artifact of university descriptions of graduate effort, but a grad student at 0.5FTE but with 100% salary support at $33K is quite confusing.

Reviewer 3:
NA

Scientific or Budget Overlap:

Description of any concerns and recommendations for follow-up and/or modifications:

Reviewer 1:
None noted

Reviewer 2:
None.

Reviewer 3:
None

ADDITIONAL RECOMMENDATIONS OR OTHER COMMENTS

Beyond the items already addressed, is there any additional advice you are willing to offer the applicant?

Reviewer 1:
None noted

Reviewer 2:
None.

Reviewer 3:
NA

Tobacco Relatedness:

Mean Score for Tobacco Relatedness: 3

Strengths:

Reviewer 1:
The proposal has high relevance to the health effects of tobacco use. It addresses a complication of smoking tobacco (stroke) and in addition, outcomes between smokers and non-smokers will be compared.

Reviewer 2:
Smoking is an independent risk factor for stroke, and many stroke survivors continue to smoke, resulting in additional complications readmissions, recurrent strokes. This study would provide vital information on the ability of the intervention to improve outcomes and quality of life in both smoking and non-smoking stroke survivors.

Reviewer 3:
The study include smoking exposure.

Tobacco Relatedness:

Weaknesses:

Reviewer 1:
None

Reviewer 2:
No substantial weaknesses.

Reviewer 3:
No clear plan to counsel for smoking cessation during period of rehab

Infrastructure (if applicable):

Strengths:

Reviewer 1:
Not applicable

Reviewer 2:
None noted

Reviewer 3:
Very good infrastructure

Infrastructure (if applicable):

Weaknesses:

Reviewer 1:
None noted

Reviewer 2:
None noted

Reviewer 3:
None
