# Supplementary material for: Respiratory Strength Training Versus Respiratory Relaxation Training in the Rehabilitation of Physical Impairment, Function, and Return to Participation After Stroke: Protocol for a Randomized Controlled Trial
Source: JMIR Res Protoc. 2024 Nov 27;13:e59749. doi: 10.2196/59749 (PMC11635318; doi:10.2196/59749)
Supplement: Multimedia Appendix 2 [file resprot_v13i1e59749_app2.pdf]

Review: Florida Department of Health James and Esther King Research Program 18-19
Proposal Number: 18-2033
Proposal Title: Augmenting a Post-Stroke Wellness Program with Respiratory Muscle Training: A Randomized Controlled Trial
Principal Investigator Name: Rose, Dorian
Proposal Mean: 3.5882

COMMENTS TO SUPPORT RATING
Beyond the items already addressed, is there any additional advice you are willing to offer the applicant?

Reviewer 1:
This study evaluates the effectiveness of respiratory muscle training intervention program to improve respiratory strength, lung health and functional mobility for 8 weeks with the intent of reducing stroke complications such as dysphagia and pneumonia leading to re-hospitalization and recurrent strokes. This study will also evaluate the impact of this program intervention on smoking among stroke patients who smoke and tolerance to exposure to smoke among non-smokers. The issues include the lack of preliminary data on the success of this program, the rate of stroke patients having issues with respiratory muscles and lack of details concerning data collection and data management (including data security) which is disconcerting.

Reviewer 2:
No comments

Reviewer 3:
Solid and potentially impactful study. Optimistic effect size from power analysis that only 60 participants are needed to obtain 80% power, but otherwise solid study

Reviewer 4:
This proposal addresses a common health problem with a high risk of poor outcomes. The study lacks a conceptual framework.

Reviewer 5:
One of the weaknesses and cause for concern is the use of a fitbit device for a research study. Has this been done before? Are they reliable enough? What is the tobacco relatedness- seemed to be secondary. Will the findings of this study be generalizable?

Reviewer 6:
No.

Reviewer 7:
Proposal addresses the important issue of how best to alter the respiratory health and quality of life of smokers and non-smokers after a stroke. Generalizability of results is questioned given the single study site. Nonetheless, the study will be able to be a model for other investigations at other sites.

Reviewer 8:
No additional comments.

Reviewer 9:
None.

Reviewer 10:
The proposal would find out if a particular new rehab treatment improved breathing after stroke, similarly in those who smoked before the stroke and those who did not. It was not clear if recruitment would select for, or track, smoking after the stroke during rehab. It was not clear if they could observe whether better post stroke lung functioning allowed pre-stroke smokers to smoke again, in contrast with worse post stroke lung functioning.

Reviewer 11:
The study is designed to answer some important questions, but it has some nonignorable weakness as rightfully pointed by the primary reviewers

Reviewer 12:
Proposal is significant, but more preliminary data (a pilot feasibility study was recommended) is needed to justify this large scale study.

Reviewer 13:
No additional comments.

Reviewer 14:
The proposed study addresses an important public health problem – stroke rehabilitation. With limited current literature and research on the effects of respiratory muscle training in stroke patients, the investigators aim to assess the effectiveness of the training program in increasing respiratory strength, lung health, and functional mobility. The investigators hold the necessary expertise and experience to conduct this clinical trial in stroke patients, with strong publication records and collaboration experiences. Perhaps one tiny nitpick would be the biostatistician with limited experience with similar randomized controlled trials and lack of prior collaboration experiences with the co-investigators. However, this study is quite innovative. Specifically, the investigators proposed the novel use of respiratory muscle training through stroke wellness clinics, with focus on patient-centered outcomes and exploratory long-term follow-up intervention strategies. The application to a chronic stroke population is highly innovative. The approach is also well thought-out, with relevant rationales to support the feasibility of the given research. The clinical trial is properly blinded, with proper inclusion/exclusion criteria, and the investigators do indeed consider relevant confounding variables. However, it is unclear to me how the investigators will complete the study in time, with recruitment and accrual leading into the fifth year, leaving little to no time for follow-up under the given grant timeframe. There has been limited discussion for potential rehabilitation intervention accuracy. As such, it is unclear how the investigators will utilize pedometers to track step counts accurately. Additionally, the study is not sufficiently powered (sample size calculations); there is little detail related to power analysis in the study. There may also be some preliminary data lacking to assure the feasibility of the proposed study.
Mainly, the proposal is well-written and address and important health concern. The investigators have recruited a wonderful research team and proposed a decent study design. However, the successful completion of this study is contingent on their ability to overcome hurdles in the inherent design of the study, such as the lack of conceptual framework for therapeutic impact after a stroke. The preliminary data are also not sufficient to assure the feasibility of the project.

Reviewer 15:
N/A

Reviewer 16:
Improving the respiratory function of stroke patients is so important, it surprises me respiratory therapy isn't a regular component of therapy already! My concerns with this study are that the statistician and graduate student coordinator may be too junior for such critical roles.

Reviewer 17:
Strengths:
1. Potential impact (exploratory aim) on understanding of complications of a history of smoking in stroke outcomes, especially in pulmonary function.
2. Excellent trained PI
3. Innovation in performing study in community based wellness program and including smoking history.
4. Study design with intermediate and long-term endpoints.
5. Innovative study as it is done in a community health wellness facility.

Weaknesses:
1. Single study site, but this is first step.
2. Relatively junior biostatistician with little experience with similar randomized trials.
3. Project coordinator is a graduate student, unusual for such a large study.
4. Smoking is an exploratory aim. More impact for stroke survivors in general.
5. Preliminary data is ok. A smaller feasibility study may be more appropriate at this stage.
6. Unclear how previous vs. current smoking history will be separated.
